# Genetic testing and reproductive decision-making in Chinese families with inherited retinal disease: a cross-sectional study

**DOI:** 10.3389/fmed.2026.1848302

**Published:** 2026-07-10

**Authors:** Victoria Y. Gu, Jingli Guo, Xuerui Zhang, Wenting Zhang, Huanyu Liu, Haodong Xiao, Dian Jiao, Jiawei Yin, Tian Tian, Peiquan Zhao

**Affiliations:** 1 Georgetown University School of Medicine, Washington, DC, United States; 2 Department of Ophthalmology, Xinhua Hospital Affiliated to Shanghai Jiao Tong University School of Medicine, Shanghai, China; 3 Department of International Health, Johns Hopkins Bloomberg School of Public Health, Baltimore, MD, United States

**Keywords:** assisted reproductive technology, genetic counseling, genetic testing, inherited retinal disease, reproductive ophthalmology

## Abstract

**Purpose:**

To identify variables associated with parental willingness toward genetic testing (GT) and subsequent reproductive (SR) intentions via natural conception (NC) or assisted reproductive technology (ART) among Chinese families with inherited retinal diseases (IRDs).

**Methods:**

Cross-sectional study of 136 children with IRDs and their biological parents at two academic referral centers in Shanghai, China (September 2023 to March 2024). Parents were surveyed on demographics, clinical characteristics, parent-reported outcome measures (ROMs) for psychosocial and economic burden, and willingness toward GT and SR. Formal genetic counseling was not available at participating centers.

**Results:**

Among 136 children [mean (SD) age, 7.10 (4.32) years; 50 (36.8%) female and their parents [mean (SD) age, 34.4 (5.8) years; 84 (61.8%) mothers], 72.1% had or were willing to undergo GT. GT willingness was positively associated with child age under 12 (OR, 1.61; *P* = 0.019), married status (OR, 5.94; *P* = 0.016), and IRD-related strife (OR, 3.61; *P* = 0.014), and negatively with parental severe vision impairment (OR, 0.19; *P* = 0.012). Child gender predicted unwillingness rationale (*P* = 0.002); parents of males cited apprehension about results, while parents of females cited cost. Most parents indicated SR unwillingness (NC: 60.3%; ART: 52.2%), with greater willingness toward ART than NC (*P* = 0.041). Both SR types were positively associated with parental vision impairment (NC: OR, 7.67; *P* = 0.029; ART: OR, 4.82; *P* = 0.046).

**Conclusion:**

Parental GT and SR decision-making was shaped by clinical and psychosocial factors, underscoring the need for genetic counseling at IRD referral centers.

## Introduction

Inherited retinal diseases (IRDs) encompass a heterogeneous group of progressive dystrophies that can lead to vision impairment and loss. These conditions arise from mutations in genes essential to retinal function, producing photoreceptor cell death and retinal pigment epithelium degeneration. Approximately 36% of the global population carries an IRD-linked mutation, one of the highest rates of carriership among human Mendelian disorders (1). Inheritance patterns and penetrance vary widely, and carrier status may remain undetected across multiple generations in the absence of clinical symptoms (1, 2). This variability extends to clinical expression: although most IRDs are congenital in origin, the age at diagnosis varies considerably, affecting both treatment efficacy and long-term visual prognosis (3, 4). The resulting clinical heterogeneity imposes substantial psychosocial and economic burden on affected families (5–7). How this burden shapes parental care-seeking and reproductive decision-making remains incompletely understood (8, 9).

Genetic testing (GT) has become integral to the management of suspected IRDs (10, 11). Beyond diagnostic confirmation and access to emerging gene therapies, molecular results provide actionable information for relatives and, in families with congenital or early-onset disease, inform reproductive planning through preimplantation genetic testing and assisted reproductive technology (ART) (8–10, 12). Yet the decision to pursue GT is shaped by considerations that extend beyond clinical utility. Genetic testing can be financially prohibitive for many families (4, 5, 12), and in the absence of curative therapy for most IRDs, the clinical utility of testing is often difficult for patients and families to appraise, underscoring the importance of structured genetic counseling in supporting informed decision-making (5, 10, 11). Molecular confirmation can also carry significant psychosocial weight. Attribution of heritable disease to one parent may precipitate intrafamilial strife, while external awareness of a hereditary condition can stigmatize the family and constrain the child's social environment. The expression and intensity of such stigma is shaped by cultural norms surrounding heritable illness, family structure, and social identity (13, 14).

The economic consequences of an IRD diagnosis for affected families are well documented, including lost income, costs associated with early age of onset, and the long-term expenses of treatment and follow-up (12, 15). Parallel evidence has established associations between vision impairment and loss (VIL) and mood and anxiety disorders (16–18), adverse quality of life (17–21), and diminished psychosocial functioning (20). A smaller but growing literature has begun to characterize the psychosocial experiences of parents and caregivers of children with eye diseases (21–24). Few studies, however, have examined how these clinical, economic, and psychosocial dimensions jointly shape parental care-seeking and reproductive decision-making (5–9).

Park and colleagues recently proposed “reproductive ophthalmology” as a conceptual framework linking ophthalmic genetics, reproductive medicine, and counseling, with the aim of encouraging multidisciplinary collaboration and rational service utilization to mitigate transmissible vision loss (8). The framework is particularly salient for IRDs, where reproductive decision-making intersects with genetic knowledge, disease burden, and sociocultural context. To date, however, most evidence on parental GT and reproductive attitudes in IRDs derives from Western cohorts operating within counseling-integrated clinical pathways, with limited reimbursement barriers to testing (5, 25). The determinants of parental decision-making in Chinese families, who navigate IRDs within distinct healthcare, economic, and cultural contexts, remain substantially underexplored, particularly with respect to the joint determinants of GT willingness and subsequent reproductive intentions via natural conception (NC) or ART.

To our knowledge, no prior study has jointly characterized the clinical, psychosocial, and economic determinants of parental GT willingness and subsequent reproductive intentions in Chinese families with IRDs. We therefore conducted a cross-sectional study of 136 patient–parent pairs at two academic referral centers in Shanghai, with two specific aims: (1) to identify clinical, psychosocial, and economic factors associated with parental willingness to pursue GT; and (2) to characterize factors associated with parental subsequent reproductive (SR) intentions via NC or ART. By situating these decisions within the reproductive ophthalmology framework, we sought to inform the development of culturally calibrated counseling pathways for IRD-affected families.

## Methods

Inherited retinal diseases impose a substantial and growing clinical burden in China, which is estimated to account for one of the largest IRD patient populations worldwide. Most published evidence on parental genetic testing and reproductive decision-making in IRDs, however, derives from Western cohorts, leaving the determinants of these decisions in Chinese families substantively underexplored. This cross-sectional study was designed to characterize those determinants within a tertiary-referral Chinese ophthalmic setting.

After demographic and clinical questions, parents were surveyed across five dimensions about their psychosocial experience of raising a child with an IRD, using ordinal scales: (1) Parental anxiety and concern about their child's future quality of life; (2) The impact of their child's IRD on personality development; (3) Experiences of peer discrimination faced by their children; (4) Social stigma encountered by the parents themselves; and (5) Marital conflict arising from the child's diagnosis. Additionally, they were asked to quantify the household COI burden and express their willingness to consider both natural and ART-based conception for future childbearing, despite the absence of guarantees against vision impact and the potential risk of inheritance (see Supplementary Appendix S1 for the full survey questionnaire). This study was conducted in accordance with the Helsinki Declaration and received Institutional Review Board approval (XHEC-D-2024-209) from the Shanghai Jiao Tong University School of Medicine.

### Instrument development

**Figure 1:**
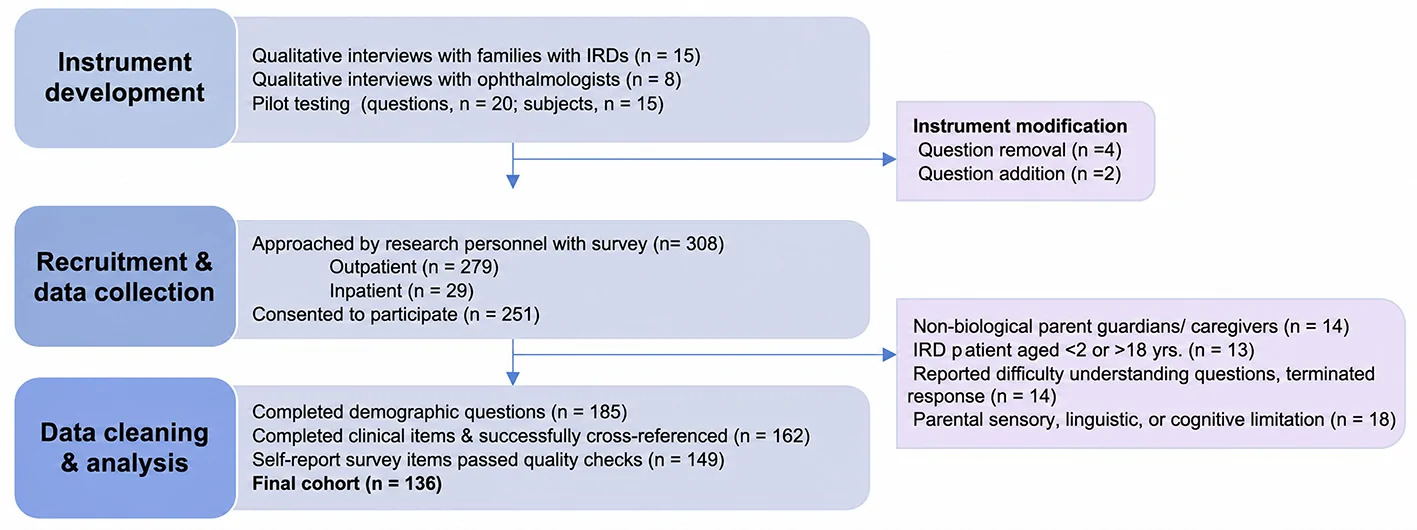
Instrument development and administration. Flow chart demonstrating survey formulation, recruitment, consent, data handling, and complete case set for Chinese familial survey of IRD-related experiences and decision-making.

Open-ended qualitative interviews were conducted with retina specialists and parents of children with diagnosed IRDs to assess research interest dimensions. Responses identified five domains of the familial IRD experience, which were integrated with literature review findings into the survey to collect parent-reported outcome measures (ROMs) on psychosocial and scenario-based questions. Pilot testing for a sample of 20 questions was conducted with 15 families and eight ophthalmologists in August 2023 to identify issues with wording or clarity. Based on pilot testing feedback, functional vision questions for parents were simplified for comprehension and eliminated for children due to non-cooperation during vision screening. The final instrument (Figure 1), finalized for data collection in September 2023, surveyed parental inclination toward gene testing, NC, and ART, as well as the psychosocial and economic dimensions of disease experience. Item wording, response-scale anchors, and clinical relevance were reviewed iteratively with retinal specialists and a subset of parent respondents during pilot testing, with revisions made to resolve ambiguities and improve cultural appropriateness for Chinese-speaking families. Formal psychometric validation of the instrument was not performed, as no validated Chinese-language measure exists for the IRD-specific domains examined; this is further discussed in the Limitations.

### Data collection

Data were collected during a 6-month period beginning in September 2023 from families attending outpatient clinic at Xinhua Hospital affiliated with Shanghai Jiao Tong University School of Medicine and Shanghai International Medical Center, two academic referral centers specializing in pediatric and vitreoretinal disease in Shanghai, China. Patients with a clinical IRD diagnosis were recruited continuously. All diagnoses were made by an attending retinal specialist on the basis of multimodal imaging, including fundus photography, optical coherence tomography, and ultra-widefield fluorescein angiography for suspected FEVR, together with detailed family history and adherence to published diagnostic criteria for each IRD subtype. Ambiguous cases were reviewed and adjudicated by consensus between two attending retinal specialists. For cases without molecular confirmation, diagnosis was supported by concordance between imaging findings, clinical phenotype, and family history. After pre-screening and confirmation of a prior IRD diagnosis, parents in either the outpatient waiting room or inpatient wards were identified as potential participants. Respondents were approached by a research personnel who detailed the scope, voluntary nature, purpose of the study, and procedures for anonymity and confidentiality to review with the researcher present. Collection did not involve any incentive measures and parents who declined participation were not reapproached. Participants were encouraged to read the form thoroughly and seek clarifications. After obtaining consent, the researcher provided the parent a physical copy of the survey for self-submission if/when completed. These procedures, including self-completion of the physical survey, recruitment by research personnel rather than the treating ophthalmologist, and the absence of participation incentives, were implemented to minimize respondent bias arising from perceived treatment-related obligation or social desirability in the clinical setting.

### Subjects

See Table 1 for inclusion and exclusion criteria.

**Table 1:** Inclusion and exclusion criteria.

Group	Category	Criterion
Inclusion	Respondent parental status	Respondent is a biological parent of a child.
	Child age and diagnosis	Children aged 2 to 18 years at the time of survey completion.
		IRD diagnosis (clinical, with or without genetic component) made or confirmed by attending ophthalmologists at Xinhua Hospital.
		Survey administration post-dated at least 2 months from the child's diagnostic confirmation.
	Respondent consent and proficiency	Parent provides informed consent for survey participation.
		Respondent is proficient in the survey's language and provides confirmation of comprehension.
Exclusion	Parental status	Non-biological and non-parent caregivers (e.g., legal guardians, grandparents, aunts, uncles).
	Diagnosis and comorbidities	Children with suspected but undiagnosed IRD.

IRD, inherited retinal disease.

### Statistical analysis

Survey findings were analyzed using descriptive statistics with listwise deletion for incomplete responses. Multivariate logistic regression was applied to identify variables associated with parental willingness to pursue genetic testing and with subsequent reproductive intentions, with results reported as odds ratios (ORs) with 95% confidence intervals and displayed as forest plots. Non-parametric Mann–Whitney *U*-tests and Kruskal–Wallis tests compared parental ROMs by child and parent traits, with Bonferroni correction applied to pairwise comparisons. Assumptions of proportional odds, observation independence, and absence of multicollinearity were checked prior to model fitting, and Q–Q plots confirmed approximate normality of regression residuals. Given the exploratory nature of the association analyses, no global multiplicity correction was applied to the logistic regression results; reported *p*-values for these analyses are therefore unadjusted and should be interpreted as hypothesis-generating. SPSS, version 23 (SPSS Inc, Chicago, IL, USA) was used for all analyses.

## Results

### Demographic and clinical characteristics

The study included 136 patient and corresponding parent pairs who met the eligibility criteria, provided informed consent, completed the survey, and whose responses passed quality checks. All demographic data can be found in Table 2. The survey had an 83.9% completion rate, while the true response rate was unknown. The cohort included 50 (36.8%) female patients and 84 (61.8%) mothers. Mean (SD) ages were 7.10 (4.32) years for patients and 34.4 (5.8) years for parents. Most parents were married [127 (93.4%)] and most had an associate or trade degree [73 (53.7%)], followed by bachelor [48 (35.3%)] and graduate degrees [15 (11.0%)]. Most patients had bilateral IRD [108 (79.4%)].

**Table 2:** Child and parent demographic and clinical characteristics (*N* = 136 patient and parent pairs).

Variable	Category	*n*	%
Child traits			
Age (years)	2 to 5 (infancy to preschool)	64	47.1%
	6 to 12 (primary school age)	56	41.2%
	13 to 18 (adolescence)	16	11.8%
Gender	Female	50	36.8%
	Male	86	63.2%
IRD diagnosis	Familial exudative vitreoretinopathy (FEVR)	117	86.0%
	Leber congenital amaurosis (LCA)	4	2.9%
	Retinitis pigmentosa (RP)	4	2.9%
	Leber hereditary optic neuropathy (LHON)	3	2.2%
	Autosomal recessive bestrophinopathy (ARB)	2	1.5%
	Best vitelliform macular dystrophy (Best disease)	2	1.5%
	X-linked retinoschisis (XLRS)	2	1.5%
	Stickler syndrome	2	1.5%
IRD laterality	Unilateral	28	20.6%
	Bilateral	108	79.4%
Parental traits			
Parent	Father	52	38.2%
	Mother	84	61.8%
Age (years)	26 to 30	30	22.1%
	31 to 35	51	37.5%
	36 to 42	31	22.8%
	Over 42	24	17.6%
Marital status	Married	127	93.4%
	Divorced, separated, or single	9	6.6%
Educational attainment	Associate or trade degree, or below	73	53.7%
	Bachelor's degree	48	35.3%
	Graduate degree	15	11.0%
Eye condition	Normal (at or above −0.6 D)	102	75.0%
	High myopia (below −0.6 D)	30	22.1%
	History of retinal detachment	7	5.1%
	Diagnosed IRD	6	4.4%

Values are presented as *n* (%); percentages are calculated from the total cohort (*N* = 136). Eye condition categories are not mutually exclusive, as parents could meet more than one criterion. D, diopter; IRD, inherited retinal disease.

Among the parents, 102 (75.0%) had normal vision, defined as a diopter measurement at or above −0.6, indicating low or no myopia. This was followed by those with high myopia, or a diopter measurement below −0.6 [30 (22.1%)], retinal detachment [7 (5.1%)], or an IRD diagnosis confirmed by physicians at either of the 2 clinical sites [6 (4.4%)]. Familial exudative vitreoretinopathy (FEVR) accounted for 117 (86.0%) of IRD cases, followed by Leber congenital amaurosis [LCA; 4 (2.9%)], retinitis pigmentosa [RP; 4 (2.9%)], Leber hereditary optic neuropathy [LHON; 3 (2.2%)], autosomal recessive bestrophinopathy [ARB; 2 (1.5%)], Best disease [2 (1.5%)], X-linked retinoschisis [XLRS; 2 (1.5%)], and Stickler syndrome [2 (1.5%)]. This diagnostic distribution reflects the referral pattern of the participating center, which receives a disproportionate number of FEVR cases compared to other IRDs.

### Parental decision-making: genetic testing

The distribution of parental inclination toward GT was as follows: 98 (72.1%) were willing or had obtained GT, 16 (11.8%) were unwilling due to apprehension toward knowing results, 14 (10.3%) were unwilling and cited cost as a barrier, and 8 (5.9%) were unwilling for other reasons (see Supplementary Table S1). Among parents who cited a desire not to know results of GT, 81% were parents of male children with an IRD. In turn, 79% of parents citing a cost barrier to testing had female children (Supplementary Figure S1).

**Figure 2:**
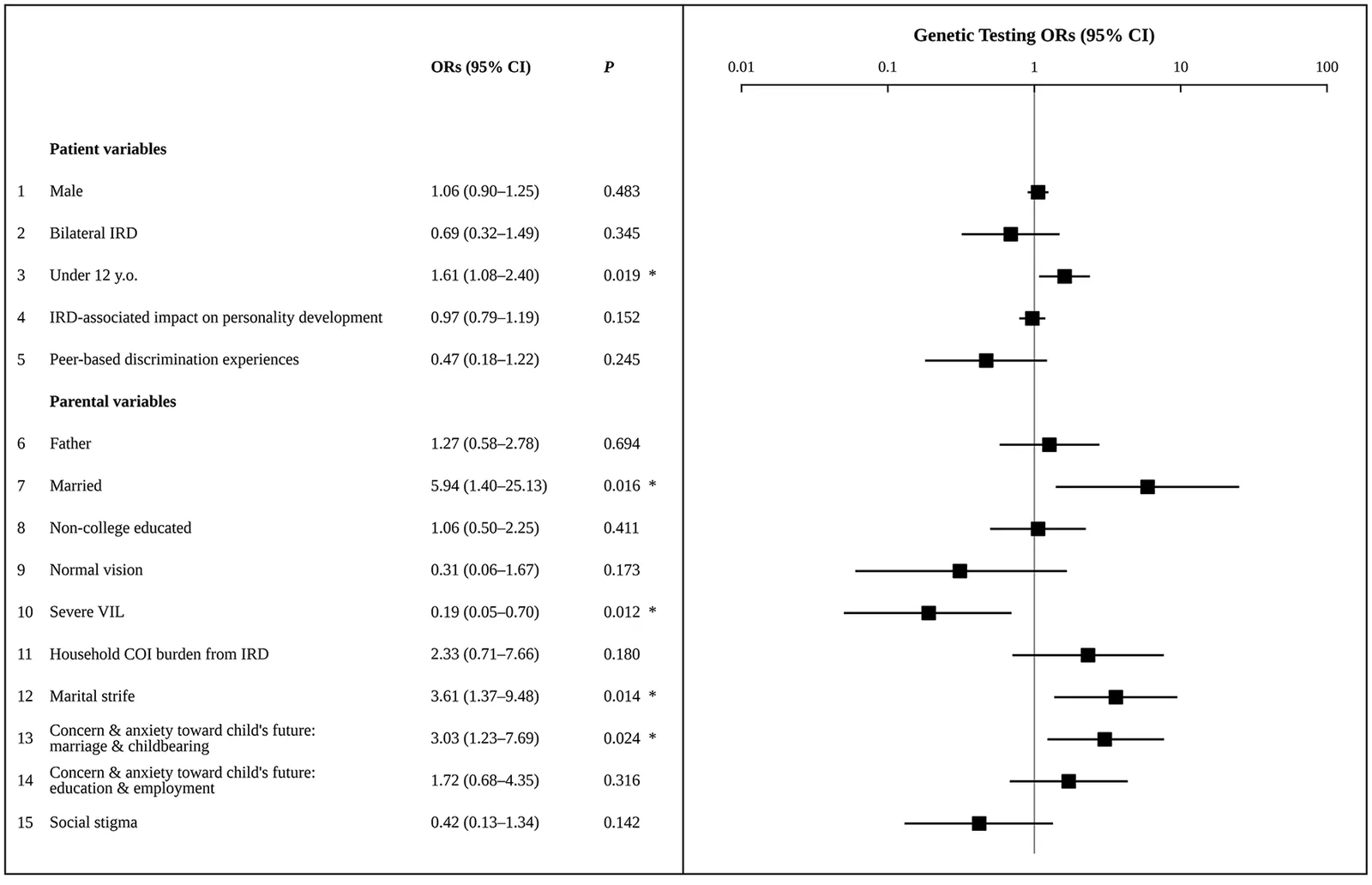
Patient traits and parent-ROMs associated with parental willingness to obtain genetic testing. Forest plot of odds ratios and 95% confidence intervals for patient and parental variables influencing the likelihood of participating in genetic testing for IRDs. Variables of interest include child age, as well as parental marital status and reported strife, vision loss, and reported concern toward their child's future quality of life. Odds ration are reported in relation to baseline categories, with significant associations indicated by an asterisk (*). OR, odds ratio; CI, confidence interval. *p < 0.05.

Chi-square analysis demonstrated that child age and parental willingness had a significant association (χ^2^ = 139.450, df = 12, *P* < 0.001), with notably higher levels of willingness observed in the infancy to preschool (2–5 years) and primary school (6–12 years) age ranges. Associations between willingness to obtain GT and patient and parent variables and ROMs are shown in Figure 2.

Parental variables significantly positively associated with testing willingness included being married (OR, 5.94 [95% CI: 1.40–25.13]; *P* = 0.016), having a child with an IRD under the age of 12 (OR, 1.61 [95% CI: 1.08–2.40]; *P* = 0.019), reporting marital strife (OR, 3.61 [95% CI: 1.37–9.48]; *P* = 0.014), and reporting concern or anxiety toward their child's future marriage and childbearing (OR, 3.03 [95% CI: 1.23–7.69]; *P* = 0.024). The only variable significantly negatively associated with willingness was parental severe vision impairment (SVI; OR, 0.19 [95% CI: 0.05–0.70]; *P* = 0.012); parental normal vision showed a non-significant association in the same direction (OR, 0.31 [95% CI: 0.06–1.67]; *P* = 0.173).

### Parental decision-making: natural and ART reproductive willingness

**Figure 3:**
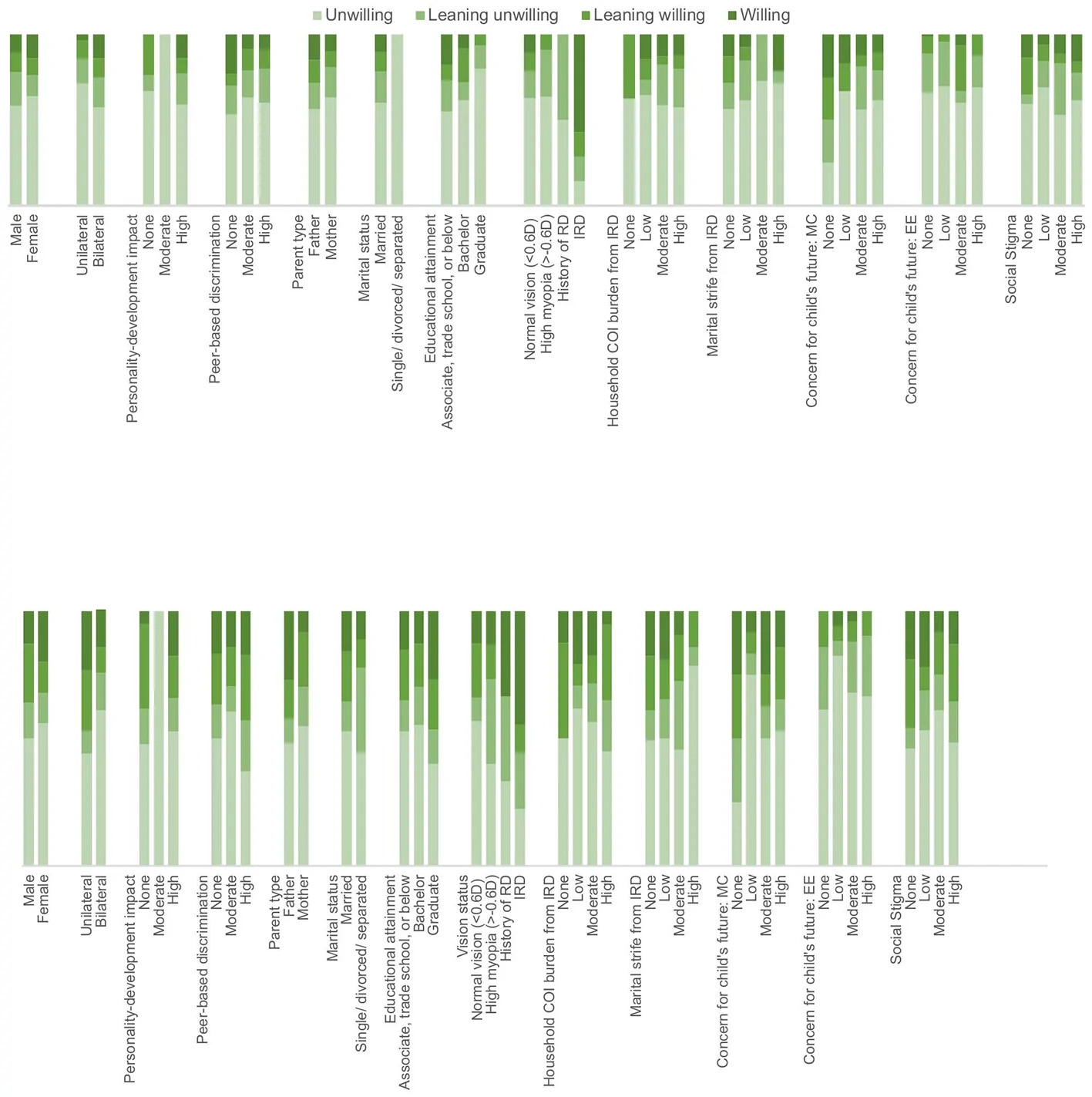
Distribution of parental reproductive willingness by child and parental characteristics among 136 Chinese families with inherited retinal disease. Stacked bars show the percentage of respondents in each willingness category (unwilling; hesitant, leaning unwilling; hesitant, leaning willing; willing) at each level of the child and parental variables examined, for natural conception **(above)** and assisted reproductive technology (ART)-based conception **(below)**. Darker shades denote greater willingness. Overall, willingness was higher for ART than for natural conception.

When asked whether they would be willing to naturally conceive of future children, 82 (60%) respondent parents were unwilling, 23 (16.9%) were hesitant but leaning unwilling, 15 (11.0%) were hesitant but leaning willing, and 16 (11.8%) were willing (see Supplementary Tables S2, S3). In turn, when asked about inclination toward subsequent childbearing with ART, without guarantee of efficacy in eliminating disease burden, 71 (52.2%) indicated unwillingness, 18 (13.2%) were hesitant/leaning unwilling, 26 (19.1%) were hesitant/leaning willing, and 21 (15.4%) were willing (see Figure 3). A paired samples *t*-test comparing willingness to have another child via NC vs. ART indicated a statistically significant difference in inclination [*t*_(132)_ = −2.066; *P* = 0.041, two-tailed]. Willingness to conceive via NC was significantly lower than via ART (mean difference −0.226, SD 1.259, 95% CI −0.442 to −0.010; paired *t*_(132)_ = −2.066, *P* = 0.041).

**Figure 4:**
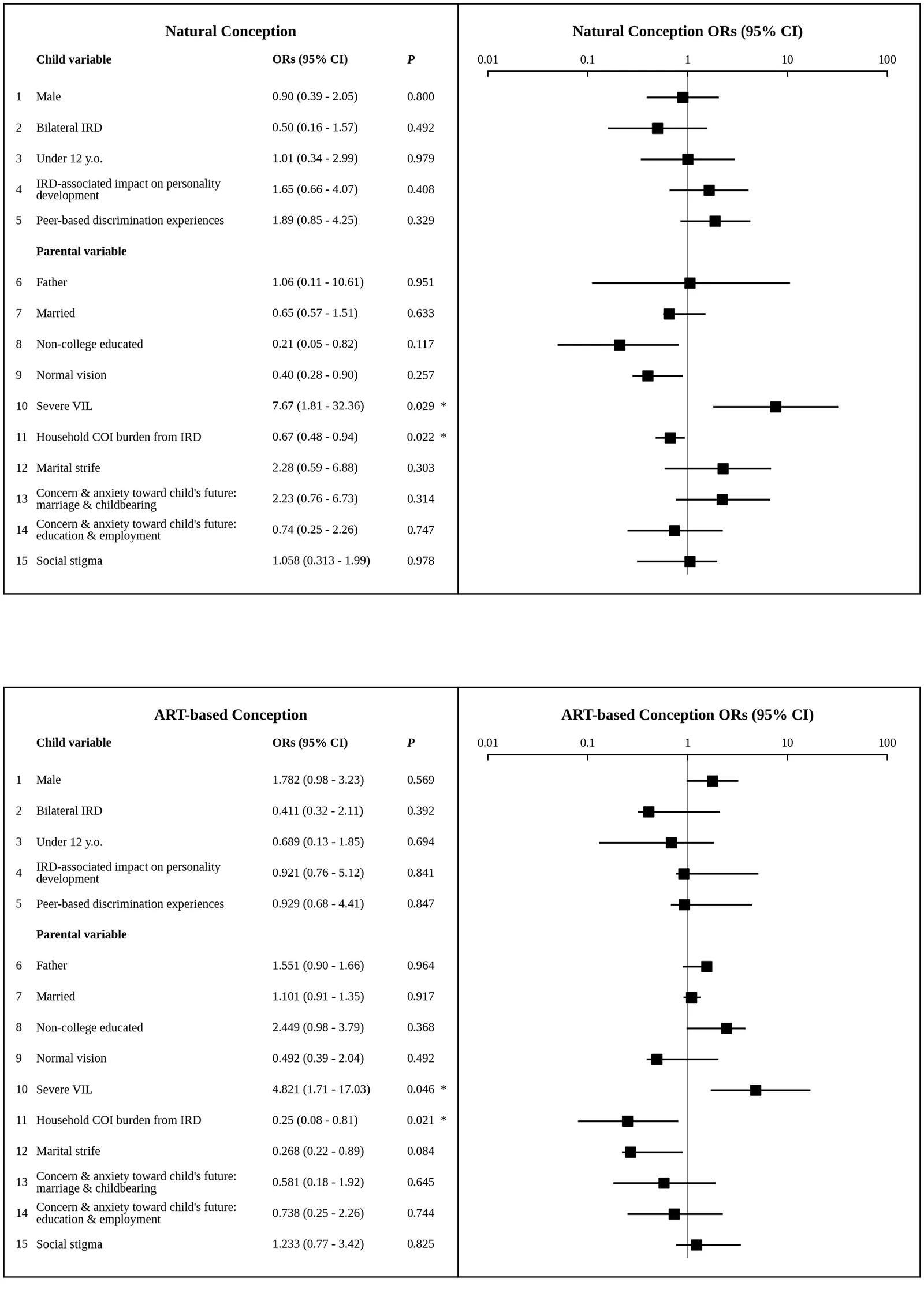
Patient traits and parent-ROMs associated with parental subsequent reproductive willingness: natural and ART-based conception. Forest plots of odds ratios and 95% confidence intervals for child and parental variables influencing the likelihood of natural conception **(above)** and assisted reproductive technology (ART)-based conception **(below)** among patients with IRDs. In the natural conception plot, notable variables include parental visual loss. In the ART-based conception plot, parental VIL is also significantly associated with ART-based conception. Other variables such as the child's age, peer-based discrimination experiences, and parental marital status show no significant associations in either plot. Odds ration are reported in relation to baseline categories, with significant associations indicated by an asterisk (*). OR, odds ratio; CI, confidence interval. *P < 0.05.

Associations between parental variables and SR willingness for both NC and ART-based conception are shown in Figure 4. Parental SVI was associated with increased subsequent willingness via both NC (OR, 7.667 [95% CI: 1.810–32.36]; *P* = 0.029) and ART-based conception (OR, 4.82 [95% CI: 1.71–17.03]; *P* = 0.046). Parental age showed a very weak negative correlation with NC willingness (Pearson: −0.080; *P* = 0.355) and a weak negative correlation with ART-based conception (Pearson: −0.178; *P* = 0.040). Among parents who indicated inclination toward subsequent reproduction and reported no or low IRD-related strife, 81.3% were amenable to NC, while 95.2% were open to using ART. Parent-reported COI also demonstrated significant negative associations with both natural (OR, 0.67 [95% CI: 0.48–0.94]; *P* = 0.022) and ART (OR, 0.25 [95% CI: 0.08–0.81]; *P* = 0.021). No patient traits nor experiences were significantly correlated with either type of SR willingness.

## Discussion

Our findings can be read through the organizing framework of reproductive ophthalmology, which situates parental decision-making about genetic testing and subsequent reproduction at the intersection of ophthalmic genetics, reproductive medicine, and counseling (8). Within this framework, three analytical dimensions emerged from our data. First, parental willingness to pursue genetic testing was shaped less by the clinical features of the child's disease than by family-level factors, including marital status, intrafamilial strife, and the parent's own vision status. Second, subsequent reproductive intentions were governed by a distinct set of determinants, dominated by parental vision impairment and perceived cost burden. Third, a consistent cost-vs.-apprehension axis distinguished unwilling parents, with cost concerns more frequently raised by parents of female children and fear of results more frequently raised by parents of male children. The sections that follow address each of these dimensions in turn, and locate the findings within both the existing IRD literature and the sociocultural context in which they were observed.

### Determinants of parental genetic testing willingness

Previous studies have reported that cohorts have expressed both positive and negative feelings toward GT, citing both the advantages of diagnostic confirmation and risk information and the disadvantages of knowledge burden and moral dilemmas around fertility (25–28). The study revealed a substantial inclination toward GT among parents, which is consistent with frequencies previously reported for heritable eye conditions (5–7).

This study identified several factors influencing parental GT willingness, primarily shaped by parental traits and ROMs. Notably, the negative association with child age emerged as the sole patient-related factor affecting parental intentions. Higher testing willingness in parents of younger children may reflect a desire to inform fertility, while lower willingness in parents of older children may suggest increased autonomy, disease adaptation, and reduced perceived benefits, underscoring the importance of reminding families with adolescent children about the benefits of GT.

Parental variables such as marriage, vision loss, and concerns about their child's future marriage/childbearing were associated with obtaining or having obtained GT. The former findings are thematically consistent with qualitative interview feedback, wherein some suggested fear of attribution and family harmony impact. The broad CI may be due to the limited number of divorced/separated parents; a larger cohort would allow for more definitive interpretations. Logistic regression further indicated marital strife as a significant predictor for testing unwillingness rationale (*P* = 0.006, high strife; *P* = 0.007, moderate strife), and higher levels of strife were associated with a greater likelihood of citing “apprehension toward knowing results.” These findings suggest that strife significantly influences parental GT intentions. Understanding the impact of marital strife on and from GT decisions can inform interventions and family counseling. Further, the association of VIL with willingness was also previously documented in a Chinese cohort with IRDs (6). Of interest, for parents with a diagnosed IRD, one-third indicated willingness, while two-thirds cited a cost burden (Supplementary Table S1). This pattern of decision-making could reflect (1) the financial constraints of existing IRD-related VIL, and/or (2) a lack of perceived testing utility when inheritance seems apparent. Qualitative interviews revealed that some parents considered subsequent childbearing to support existing family members with VIL. A follow-up study could review parents' fundus imaging findings to assess if other, non-definitive suggestions of parental attribution impact subsequent GT inclination.

Regarding psychosocial ROMs, most parents expressed high degrees of anxiety and concern toward their children's future QOL, and these experiences have shown correlations with parental health-seeking behavior (29). Further research on GT attitudes in families with other congenital inherited diseases would provide insight into culture-specific decision-making. Parents reported high degrees of both economic and psychosocial burden, though not associated with GT willingness, suggesting high caregiver strain from genetic attribution as described in other populations (25, 27, 28).

### Cultural context and comparison with Western cohorts

The overall rate of GT willingness in our cohort (72.1%) is broadly concordant with uptake figures reported in Western IRD populations, though the structural and motivational determinants of willingness diverge in informative ways. In Western cohorts, testing uptake has been principally framed around diagnostic resolution, gene-therapy eligibility, and inheritance clarification for adult family members, supported by subsidized testing and counseling-integrated clinical pathways (5, 25, 30, 31). In our cohort, by contrast, willingness was more strongly shaped by marital status, IRD-related family strife, and anxiety about the child's future marriage and childbearing prospects, reflecting the centrality of familial continuity and the social consequences of heritable disease within Chinese cultural norms rather than a deficit in attitudinal support for testing. The gendered pattern of unwillingness observed here, in which cost was cited predominantly by parents of female children and apprehension about results by parents of male children, is consistent with documented patterns of gendered health expenditure and pediatric healthcare utilization in Chinese families (32, 33), and with US evidence that racial and out-of-pocket cost barriers independently reduce the likelihood of definitive genetic diagnosis (34), indicating that cost-related barriers manifest in structurally distinct ways across healthcare systems, with delayed genetic testing itself associated with higher cumulative healthcare resource utilization in US cohorts (34, 35).

Cross-cultural differences are most pronounced in reproductive decision-making. Western literature on reproductive planning in heritable disease has centered largely on the acceptability of preimplantation genetic testing for monogenic disorders (PGT-M) and prenatal diagnosis as mechanisms for avoiding affected offspring, framed within Park et al.'s conceptualization of reproductive ophthalmology (8) and recent work by Redgrave and McNeill (36) and Gregersen et al. (9) on heritable vision-loss cohorts. The predominant reluctance toward subsequent reproduction observed in our cohort (60% unwilling for natural conception, 52% for ART) is unlikely to reflect a simple attitudinal difference; it more plausibly reflects the interaction of disease burden with China's current demographic and economic climate, including declining fertility, rising costs of childrearing, and limited integration of reproductive genetic counseling into ophthalmic care. Parallels observed in other Asia-Pacific and Asian diaspora cohorts, including family-centered testing decisions, expectations of directive counseling, and attention to disease attribution and marriageability, reinforce the interpretation that cultural context, rather than attitudinal deficit, shapes these decisions (37–39).

Our findings are therefore best understood as complementary to, rather than directly comparable with, Western IRD cohorts. The absolute level of GT willingness is similar, but determinants, rationales, and downstream reproductive implications differ in ways that reflect both cultural context and the structural availability of counseling and subsidized testing. Disentangling culturally mediated drivers from system-level constraints will require multi-center collaboration. The recently established Asia Pacific Network for Inherited Eye Diseases (APIED), which documents both the heterogeneity of IRD clinical practice and persistent regional scarcity of trained genetic counselors (40, 41), offers a suitable platform for future comparative studies of decision-making under varying counseling and reimbursement conditions.

### Determinants of subsequent reproductive intentions

Reproductive willingness is an increasingly topical subject for Chinese families amidst population aging and rising living costs. While no association by parent type was found across either category of willingness, paternal willingness nearly doubled (15% vs. 27%) for ART compared to NC, while maternal willingness exhibited negligible variation (10% vs. 8%; Supplementary Table S2). Subsequent research may help delineate SR willingness disparities between IRDs and non-heritable ophthalmic conditions. Additionally, while the cosmetic and psychosocial consequences of pediatric eye disease are well-documented (16–21), and most parents reported high degrees of psychosocial burden, no associations with SR decision making were found (Figure 4).

The association between VIL and increased SR willingness may suggest that parents who live with VIL, and/or have internalized inheritance risk, are more receptive toward future children. In turn, the findings of a near-significant association between parental “normal vision” and reduced SR willingness indicate that parents without VIL may perceive a higher risk or burden associated with another child, potentially yielding reluctance toward fertility (28, 29).

Although parent-reported COI burden was not associated with GT willingness (Figure 2), it was significantly and negatively associated with subsequent reproductive willingness via both NC and ART (Figure 4), indicating that perceived economic burden weighed more heavily on reproductive intentions than on testing decisions. Still, these findings underscore the cost burden of rare ophthalmic disease (21, 30, 42–44), vision loss (45), genetic interventions (28, 42), and non-coverage (30, 43, 44). Further, the difference in parents' rationale for testing unwillingness suggests gendering in decision-making. Logistic regression indicated that child gender is a meaningful predictor of the reason for unwillingness to test (*P* = 0.002). Specifically, parents of female children were more likely to cite “cost burden” (β = 2.07, SE = 0.68, *Z* = 3.04), while parents of male children were more likely to cite “fear of results.” These findings may build on other studies that have reported greater health expenditure elasticity for girls (32) and gendered differences in pediatric healthcare utilization (33).

While these findings underscore some previously reported findings on GT willingness, to our knowledge, this is the first study to assess SR intentions of IRD-affected families. As IRD biotechnologies advance, families need to be educated on options and use-cases (28, 42). These findings highlight subgroup trends in decision making, as well as the need for consultation methods that address IRD's impact on family cohesion. Critically, findings on reproductive ophthalmology decision-making seek to mitigate VIL by extent and volume (5, 25), rather than encouraging explicit decisions.

### Limitations

Our study design and setting introduce certain limitations that warrant consideration. First, formal genetic counseling services were not available at either participating center during the study period. Parental responses therefore reflect decision-making in the absence of structured pre-test risk communication, which likely contributed to variability in how inheritance, testing utility, and reproductive implications were understood, and which limits direct comparison with cohorts recruited from counseling-integrated clinics. Second, IRD diagnoses were based on clinical assessment rather than universal molecular confirmation. Third, the predominance of FEVR cases reflects the referral role of Xinhua Hospital as a tertiary FEVR center, which limits the representativeness of our cohort relative to the broader IRD population. Fourth, although the survey completion rate among consenting participants was 83.9%, the true response rate could not be calculated because the total number of eligible families approached during the recruitment period was not systematically logged at both sites; non-response bias of indeterminate direction therefore cannot be excluded. Fifth, the survey relied on parent-reported outcome measures captured via purpose-built ordinal Likert-type items rather than previously validated instruments, as no Chinese-language instrument has been psychometrically validated for the IRD-specific domains of parental psychosocial burden and reproductive intention examined here. Parent-reported measures may also be subject to social desirability or extreme response bias. Lastly, the study employed a single-respondent, family-unit design, with surveys completed by the primary caregiver present at the clinic visit. While this approach was elected for consistency, it may not capture differences in perception between both caregivers within a family.

## Conclusion

In this cross-sectional study of 136 Chinese families affected by IRDs, parental willingness to pursue genetic testing and to consider subsequent reproduction was shaped by largely distinct sets of determinants. Marital status, intrafamilial strife, and parental severe vision impairment were the dominant predictors of testing willingness, while parental vision impairment and perceived cost burden most strongly influenced reproductive intentions. A consistent gendered pattern emerged among parents declining testing, with cost concerns more frequently raised by parents of female children and apprehension about results by parents of male children. These findings, situated within the framework of reproductive ophthalmology, suggest that parental decision-making in this population is driven less by clinical features of the child's disease than by family-level and contextual factors. Integration of accessible genetic counseling into IRD referral pathways, attentive to the cultural and economic context in which decisions are made, may help address the heterogeneity in parental responses observed here. Confirmation in independent Chinese cohorts and in counseling-integrated settings is warranted.

## Data Availability

The raw data supporting the conclusions of this article will be made available by the authors, without undue reservation.

## Author's note

Meeting presentation: Presented as a poster at the American Society of Retina Specialists (ASRS) 2024 Annual Meeting, Stockholm, Sweden.
