# Correction: Genetic testing and reproductive decision-making in Chinese families with inherited retinal disease: a cross-sectional study

**DOI:** 10.3389/fmed.2026.2009714

**Published:** 2026-10-16

**Authors:** Victoria Y. Gu, Jingli Guo, Xuerui Zhang, Wenting Zhang, Huanyu Liu, Haodong Xiao, Dian Jiao, Jiawei Yin, Tian Tian, Peiquan Zhao

**Affiliations:** 1 Georgetown University School of Medicine, Washington, DC, United States; 2 Department of Ophthalmology, Xinhua Hospital Affiliated to Shanghai Jiao Tong University School of Medicine, Shanghai, China; 3 Department of International Health, Johns Hopkins Bloomberg School of Public Health, Baltimore, MD, United States

**Keywords:** assisted reproductive technology, genetic counseling, genetic testing, inherited retinal disease, reproductive ophthalmology

The funders National Natural Science Foundation of China, 82000904, and Shanghai Science and Technology Innovation Action Plan (Medical Innovation Research Project), 23Y11901200, to Tian Tian were erroneously omitted.

The correct Funding statement is:

“The author(s) declare that financial support was received for the research and/or publication of this article. This work was supported by the National Key R&D Program of China (grant no. 2024YFC2510900), the National Natural Science Foundation of China (grant nos. 82171069 and 82000904), and the Shanghai Science and Technology Innovation Action Plan (Medical Innovation Research Project) (grant nos. 23Y11901200 and 22Y11910200).”

The original version of this article has been updated.

