# Supplementary material for: Genetic testing and reproductive decision-making in Chinese families with inherited retinal disease: a cross-sectional study
Source: Front Med (Lausanne). 2026 Jul 10;13:1848302. doi: 10.3389/fmed.2026.1848302 (PMC13395770; doi:10.3389/fmed.2026.1848302)
Supplement: Supplementary file 1 [file Supplementary_file_1.docx]

**Supplemental Data**

**Keywords:** inherited retinal disease, genetic testing, reproductive ophthalmology, assisted reproductive technology, genetic counselling

eFigure 1. Parental attitudes toward genetic testing for IRD, by child variables (N = 136)

eTable 1. Parental attitudes toward genetic testing for IRD, by demographic, clinical, and ROM variables (N = 136)

eTable 2. Parental subsequent reproductive willingness toward natural vs ART-based conception for IRD, by demographic and clinical variables (N = 136)

eTable 3. Parental subsequent reproductive willingness toward natural vs ART-based conception for IRD, by ROM variables (N = 136)

eAppendix 1. Survey Questionnaire (English Translation)

**eFigure 1.** Parental attitudes toward genetic testing for IRD, by child variables (N = 136)


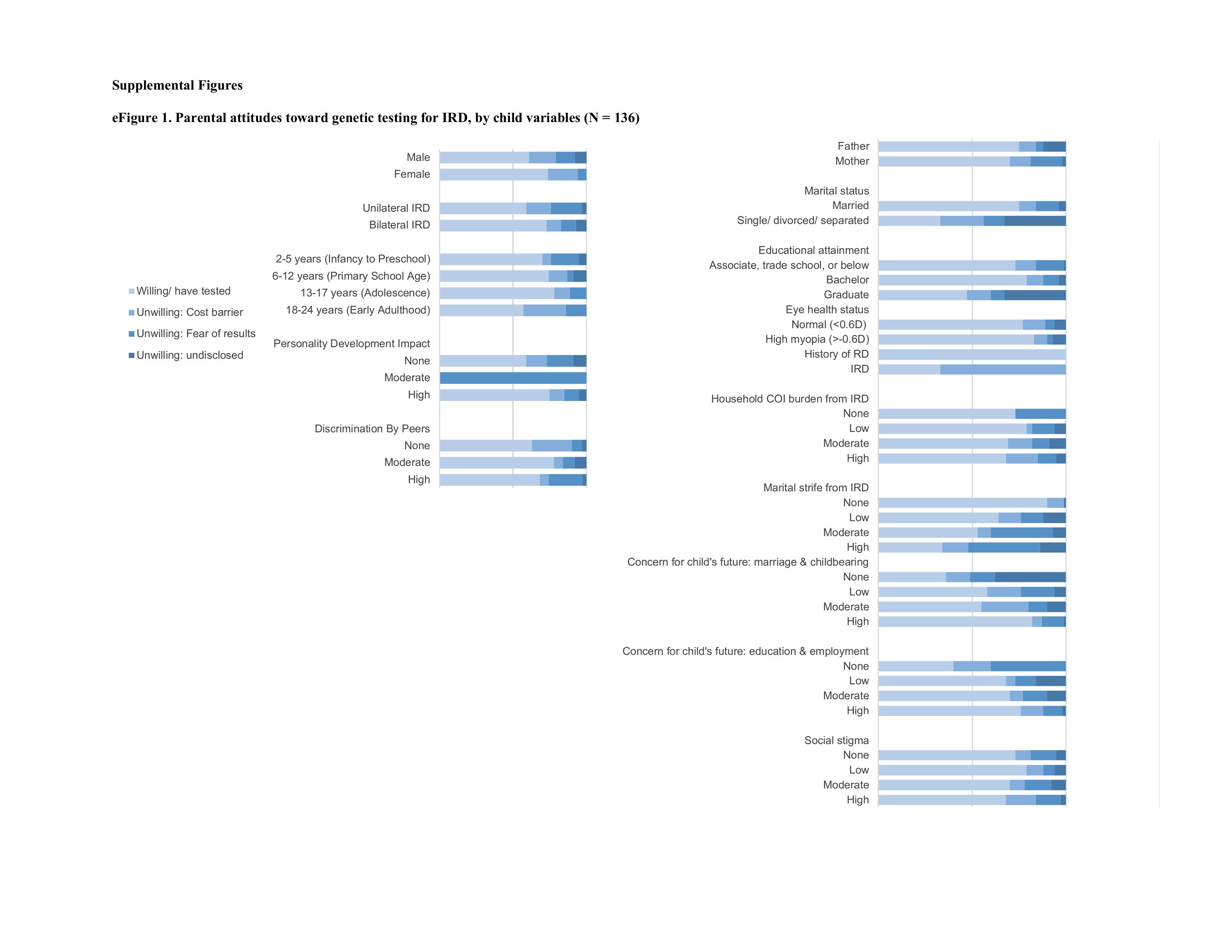


**eTable 1.** Parental attitudes toward genetic testing for IRD, by demographic, clinical, and ROM variables (N = 136)

*Values are presented as n (%).*

|  | **Willing/tested (n = 98)** | **Cost barrier (n = 14)** | **Fear of results (n = 16)** | **Undisclosed (n = 8)** |
| --- | --- | --- | --- | --- |
|  | **n (%)** | **n (%)** | **n (%)** | **n (%)** |
| **Patient variables** |  |  |  |  |
| **Gender** |  |  |  |  |
| Male | 60 (70%) | 3 (21%) | 13 (15%) | 8 (9%) |
| Female | 38 (76%) | 11 (21%) | 3 (6%) | 0 (0%) |
| **Disease laterality** |  |  |  |  |
| Unilateral | 20 (71%) | 3 (11%) | 5 (18%) | 0 (0%) |
| Bilateral | 78 (72%) | 11 (10%) | 11 (10%) | 8 (7%) |
| **Age** |  |  |  |  |
| 2-5 y (Infancy to Preschool) | 45 (70%) | 4 (6%) | 12 (19%) | 3 (5%) |
| 6-12 y (Primary School) | 42 (75%) | 7 (13%) | 2 (4%) | 5 (9%) |
| 13-17 y (Adolescence) | 7 (78%) | 1 (11%) | 1 (11%) | 0 (0%) |
| 18-24 y (Early Adulthood) | 4 (57%) | 2 (29%) | 1 (14%) | 0 (0%) |
| **Personality-development impact** |  |  |  |  |
| None | 13 (59%) | 3 (14%) | 4 (18%) | 2 (9%) |
| Moderate | 0 (0%) | 0 (0%) | 1 (100%) | 0 (0%) |
| High | 85 (75%) | 11 (10%) | 11 (10%) | 6 (5%) |
| **Peer-based discrimination** |  |  |  |  |
| None | 19 (63%) | 8 (27%) | 2 (7%) | 1 (3%) |
| Moderate | 55 (77%) | 4 (6%) | 6 (8%) | 6 (8%) |
| High | 24 (69%) | 2 (6%) | 8 (23%) | 1 (3%) |
| **Parental variables** |  |  |  |  |
| **Parent type** |  |  |  |  |
| Father | 39 (75%) | 5 (10%) | 2 (4%) | 6 (12%) |
| Mother | 59 (70%) | 9 (11%) | 14 (17%) | 2 (2%) |
| **Marital status** |  |  |  |  |
| Married | 95 (75%) | 12 (9%) | 15 (12%) | 5 (4%) |
| Single/divorced/separated | 3 (33%) | 2 (22%) | 1 (11%) | 3 (33%) |
| **Educational attainment** |  |  |  |  |
| Associate/trade school or below | 53 (73%) | 8 (11%) | 11 (15%) | 1 (1%) |
| Bachelor | 38 (79%) | 4 (8%) | 4 (8%) | 2 (4%) |
| Graduate | 7 (47%) | 2 (13%) | 1 (7%) | 5 (33%) |
| **Eye health status** |  |  |  |  |
| Normal (<0.6 D) | 79 (77%) | 12 (12%) | 5 (5%) | 6 (6%) |
| High myopia (>-0.6 D) | 25 (83%) | 2 (7%) | 1 (3%) | 2 (7%) |
| History of RD | 7 (100%) | 0 (0%) | 0 (0%) | 0 (0%) |
| IRD | 2 (33%) | 4 (67%) | 0 (0%) | 0 (0%) |
| **Household COI burden from IRD** |  |  |  |  |
| None | 11 (73%) | 0 (0%) | 4 (27%) | 0 (0%) |
| Low | 26 (79%) | 1 (3%) | 4 (12%) | 2 (6%) |
| Moderate | 32 (70%) | 6 (13%) | 4 (9%) | 4 (9%) |
| High | 29 (69%) | 7 (17%) | 4 (10%) | 2 (5%) |
| **Marital strife from IRD** |  |  |  |  |
| None | 66 (89%) | 7 (9%) | 0 (0%) | 1 (1%) |
| Low | 17 (65%) | 3 (12%) | 3 (12%) | 3 (12%) |
| Moderate | 8 (53%) | 1 (7%) | 5 (33%) | 1 (7%) |
| High | 7 (33%) | 3 (14%) | 8 (38%) | 3 (14%) |
| **Concern: marriage & childbearing** |  |  |  |  |
| None | 3 (38%) | 1 (13%) | 1 (13%) | 3 (38%) |
| Low | 10 (59%) | 3 (18%) | 3 (18%) | 1 (6%) |
| Moderate | 11 (55%) | 5 (25%) | 2 (10%) | 2 (10%) |
| High | 74 (81%) | 5 (5%) | 10 (11%) | 2 (2%) |
| **Concern: education & employment** |  |  |  |  |
| None | 2 (40%) | 1 (20%) | 2 (40%) | 0 (0%) |
| Low | 13 (68%) | 1 (5%) | 2 (11%) | 3 (16%) |
| Moderate | 21 (70%) | 2 (7%) | 4 (13%) | 3 (10%) |
| High | 62 (76%) | 10 (12%) | 8 (10%) | 2 (2%) |
| **Social stigma** |  |  |  |  |
| None | 27 (73%) | 3 (8%) | 5 (14%) | 2 (5%) |
| Low | 25 (78%) | 3 (9%) | 2 (6%) | 2 (6%) |
| Moderate | 25 (69%) | 3 (8%) | 5 (14%) | 3 (8%) |
| High | 21 (68%) | 5 (16%) | 4 (13%) | 1 (3%) |

**eTable 2.** Parental subsequent reproductive willingness toward natural vs ART-based conception for IRD, by demographic and clinical variables (N = 136)

*Values are presented as n (%). NC, natural conception; ART, assisted reproductive technology.*

|  | **Unwilling** |  | **Hesitant, leaning unwilling** |  | **Hesitant, leaning willing** |  | **Willing** |  |
| --- | --- | --- | --- | --- | --- | --- | --- | --- |
|  | **NC** | **ART** | **NC** | **ART** | **NC** | **ART** | **NC** | **ART** |
|  | *n = 82* | *n = 71* | *n = 23* | *n = 18* | *n = 15* | *n = 26* | *n = 16* | *n = 21* |
| **Patient variables** |  |  |  |  |  |  |  |  |
| **Gender** |  |  |  |  |  |  |  |  |
| Male | 50 (58%) | 43 (50%) | 17 (20%) | 12 (14%) | 10 (12%) | 20 (23%) | 9 (10%) | 11 (13%) |
| Female | 32 (64%) | 28 (56%) | 6 (12%) | 6 (12%) | 5 (10%) | 6 (12%) | 7 (14%) | 10 (20%) |
| **Disease laterality** |  |  |  |  |  |  |  |  |
| Unilateral | 20 (71%) | 13 (46%) | 4 (14%) | 2 (7%) | 3 (11%) | 8 (29%) | 1 (4%) | 5 (18%) |
| Bilateral | 62 (57%) | 58 (54%) | 19 (18%) | 16 (15%) | 12 (11%) | 18 (17%) | 15 (14%) | 16 (15%) |
| **Parental variables** |  |  |  |  |  |  |  |  |
| **Parent type** |  |  |  |  |  |  |  |  |
| Father | 30 (58%) | 25 (48%) | 8 (15%) | 5 (10%) | 7 (13%) | 8 (15%) | 8 (15%) | 14 (27%) |
| Mother | 52 (62%) | 46 (55%) | 15 (18%) | 13 (15%) | 8 (10%) | 18 (21%) | 8 (10%) | 7 (8%) |
| **Marital status** |  |  |  |  |  |  |  |  |
| Married | 76 (60%) | 67 (53%) | 23 (18%) | 15 (12%) | 15 (12%) | 25 (20%) | 16 (13%) | 20 (16%) |
| Single/divorced/separated | 6 (40%) | 4 (44%) | 0 (0%) | 3 (33%) | 0 (0%) | 1 (11%) | 0 (0%) | 1 (11%) |
| **Educational attainment** |  |  |  |  |  |  |  |  |
| Associate/trade or below | 41 (55%) | 39 (53%) | 16 (22%) | 9 (12%) | 7 (9%) | 15 (20%) | 13 (18%) | 11 (15%) |
| Bachelor | 29 (62%) | 26 (55%) | 5 (11%) | 7 (15%) | 7 (15%) | 8 (17%) | 2 (4%) | 6 (13%) |
| Graduate | 12 (80%) | 6 (40%) | 2 (13%) | 2 (13%) | 1 (7%) | 3 (20%) | 1 (7%) | 4 (27%) |
| **Eye health status** |  |  |  |  |  |  |  |  |
| Normal (<0.6 D) | 73 (63%) | 62 (57%) | 18 (16%) | 10 (9%) | 13 (11%) | 23 (21%) | 12 (10%) | 14 (13%) |
| High myopia (>-0.6 D) | 7 (64%) | 6 (40%) | 3 (27%) | 5 (33%) | 1 (9%) | 2 (13%) | 0 (0%) | 2 (13%) |
| History of RD | 1 (50%) | 1 (33%) | 1 (50%) | 1 (33%) | 0 (0%) | 0 (0%) | 0 (0%) | 1 (33%) |
| IRD | 1 (14%) | 2 (22%) | 1 (14%) | 2 (22%) | 1 (14%) | 1 (11%) | 4 (57%) | 4 (44%) |

**eTable 3.** Parental subsequent reproductive willingness toward natural vs ART-based conception for IRD, by ROM variables (N = 136)

*Values are presented as n (%). NC, natural conception; ART, assisted reproductive technology.*

|  | **Unwilling** |  | **Hesitant, leaning unwilling** |  | **Hesitant, leaning willing** |  | **Willing** |  |
| --- | --- | --- | --- | --- | --- | --- | --- | --- |
|  | **NC** | **ART** | **NC** | **ART** | **NC** | **ART** | **NC** | **ART** |
|  | *n = 82* | *n = 71* | *n = 23* | *n = 18* | *n = 15* | *n = 26* | *n = 16* | *n = 21* |
| **Patient experiences (parent-reported)** |  |  |  |  |  |  |  |  |
| **Personality-development impact** |  |  |  |  |  |  |  |  |
| None | 14 (67%) | 10 (48%) | 2 (10%) | 3 (14%) | 5 (24%) | 7 (33%) | 0 (0%) | 1 (5%) |
| Moderate | 1 (100%) | 1 (100%) | 0 (0%) | 0 (0%) | 0 (0%) | 0 (0%) | 0 (0%) | 0 (0%) |
| High | 67 (59%) | 60 (53%) | 21 (18%) | 15 (13%) | 10 (9%) | 19 (17%) | 16 (14%) | 20 (18%) |
| **Peer-based discrimination** |  |  |  |  |  |  |  |  |
| None | 16 (53%) | 15 (50%) | 5 (17%) | 4 (13%) | 2 (7%) | 6 (20%) | 7 (23%) | 5 (17%) |
| Moderate | 45 (63%) | 43 (61%) | 11 (15%) | 7 (10%) | 9 (13%) | 11 (15%) | 6 (8%) | 10 (14%) |
| High | 21 (60%) | 13 (37%) | 7 (20%) | 7 (20%) | 4 (11%) | 9 (26%) | 3 (9%) | 6 (17%) |
| **Parental experience (self-reported)** |  |  |  |  |  |  |  |  |
| **Household COI burden from IRD** |  |  |  |  |  |  |  |  |
| None | 10 (63%) | 8 (50%) | 0 (0%) | 0 (0%) | 6 (38%) | 6 (38%) | 0 (0%) | 2 (13%) |
| Low | 22 (65%) | 21 (62%) | 3 (9%) | 3 (9%) | 4 (12%) | 3 (9%) | 5 (15%) | 7 (21%) |
| Moderate | 27 (59%) | 26 (57%) | 11 (24%) | 7 (15%) | 2 (4%) | 5 (11%) | 6 (13%) | 8 (17%) |
| High | 23 (58%) | 18 (45%) | 9 (23%) | 8 (20%) | 3 (8%) | 12 (30%) | 5 (13%) | 4 (10%) |
| **Marital strife from IRD** |  |  |  |  |  |  |  |  |
| None | 48 (56%) | 42 (49%) | 13 (15%) | 10 (12%) | 13 (15%) | 18 (21%) | 11 (13%) | 15 (18%) |
| Low | 16 (62%) | 13 (50%) | 6 (23%) | 4 (15%) | 2 (8%) | 4 (15%) | 2 (8%) | 5 (19%) |
| Moderate | 8 (73%) | 5 (45%) | 3 (27%) | 3 (27%) | 0 (0%) | 2 (18%) | 0 (0%) | 1 (9%) |
| High | 10 (71%) | 11 (79%) | 1 (7%) | 1 (7%) | 0 (0%) | 2 (14%) | 3 (21%) | 0 (0%) |
| **Anxiety & concern for child’s QOL** |  |  |  |  |  |  |  |  |
| None | 1 (25%) | 1 (25%) | 1 (25%) | 1 (25%) | 1 (25%) | 1 (25%) | 1 (25%) | 1 (25%) |
| Low | 8 (67%) | 9 (75%) | 0 (0%) | 1 (8%) | 2 (17%) | 1 (8%) | 2 (17%) | 1 (8%) |
| Moderate | 9 (56%) | 8 (50%) | 4 (25%) | 2 (13%) | 1 (6%) | 2 (13%) | 2 (13%) | 4 (25%) |
| High | 64 (62%) | 54 (52%) | 18 (17%) | 13 (13%) | 11 (11%) | 21 (20%) | 11 (11%) | 15 (14%) |
| **Social stigma** |  |  |  |  |  |  |  |  |
| None | 22 (59%) | 17 (46%) | 2 (5%) | 3 (8%) | 8 (22%) | 10 (27%) | 5 (14%) | 7 (19%) |
| Low | 22 (69%) | 17 (53%) | 5 (16%) | 5 (16%) | 3 (9%) | 3 (9%) | 2 (6%) | 7 (22%) |
| Moderate | 19 (53%) | 22 (61%) | 11 (31%) | 5 (14%) | 2 (6%) | 6 (17%) | 4 (11%) | 3 (8%) |
| High | 19 (61%) | 15 (48%) | 5 (16%) | 5 (16%) | 2 (6%) | 7 (23%) | 5 (16%) | 4 (13%) |

**eAppendix 1.** Survey Questionnaire (English Translation)

*The following questionnaire was originally administered in Simplified Chinese. This English translation is provided for reviewer and reader reference. Response options are listed beneath each item.*

**Domain 1: Parental Concern for Child’s Future Quality of Life**

**1.** Given your child’s inherited retinal disease (IRD) diagnosis, which aspect of your child’s future are you most concerned about?

a) Marriage and childbearing

b) Education

c) Employment

d) Other (please specify): _______________

**2.** Please rate your level of concern regarding your child’s future marriage and childbearing prospects:

a) Not concerned

b) Mildly concerned

c) Moderately concerned

d) Highly concerned

**3.** Please rate your level of concern regarding your child’s future educational prospects:

a) Not concerned

b) Mildly concerned

c) Moderately concerned

d) Highly concerned

**4.** Please rate your level of concern regarding your child’s future employment prospects:

a) Not concerned

b) Mildly concerned

c) Moderately concerned

d) Highly concerned

**Domain 2: Impact of IRD on Child’s Personality Development**

**5.** Do you believe that your child’s IRD-related vision impairment has affected his or her personality development?

a) No impact

b) Mild impact

c) Moderate impact

d) Significant impact

**Domain 3: Peer Discrimination Experiences**

**6.** Has your child experienced peer-based discrimination related to their IRD (eg, due to appearance or visual function)?

a) No discrimination experienced

b) Moderate discrimination experienced

c) Significant discrimination experienced

d) Unknown

**Domain 4: Parental Social Stigma**

**7.** Do you mind others knowing that your child has an IRD?

a) I do not mind

b) I mind slightly

c) I somewhat mind

d) I mind considerably

**Domain 5: Marital and Family Harmony**

**8.** Has your child’s IRD diagnosis or management of care affected your family harmony?

a) No impact

b) Mild impact

c) Moderate impact

d) Significant impact

**9.** If maternal inheritance is suggested from genetic testing, has this affected your marital harmony?

a) No impact

b) Mild impact

c) Moderate impact

d) Significant impact

e) Not applicable

**10.** If paternal inheritance is suggested from genetic testing, has this affected your marital harmony?

a) No impact

b) Mild impact

c) Moderate impact

d) Significant impact

e) Not applicable

**Genetic Testing Willingness**

**11.** When your physician recommends genetic testing to identify the causative gene for your child’s suspected IRD, are you willing to proceed? If not, please indicate the primary reason:

a) Willing

b) Unwilling — apprehension about learning the results

c) Unwilling — cost barrier

d) Unwilling — other (please specify): _______________

**Subsequent Reproductive Willingness**

**12.** Are you willing to have another child through natural conception (ie, without assisted reproductive technologies)?

a) Willing

b) Hesitant, leaning toward willing

c) Hesitant, leaning toward unwilling

d) Unwilling

**13.** Considering that existing assisted reproductive technologies cannot fully guarantee the elimination of heritable disease, are you willing to have another child through ART?

a) Willing

b) Hesitant, leaning toward willing

c) Hesitant, leaning toward unwilling

d) Unwilling

**Cost of Illness**

**14.** Please estimate the total cost of IRD-related treatment expenses incurred to date:

a) Less than ¥10,000

b) ¥10,000–¥30,000

c) ¥30,000–¥50,000

d) ¥50,000–¥100,000

e) ¥100,000–¥150,000

f) ¥150,000 or more (please specify): _______________

**15.** Please rate the burden of IRD-related expenses on your household’s financial situation:

a) No burden

b) Mild burden

c) Moderate burden

d) Significant burden

*Abbreviations: ART, assisted reproductive technology; IRD, inherited retinal disease; QOL, quality of life.*
